# Heme as Possible Contributing Factor in the Evolvement of Shiga-Toxin *Escherichia coli* Induced Hemolytic-Uremic Syndrome

**DOI:** 10.3389/fimmu.2020.547406

**Published:** 2020-12-22

**Authors:** Kioa L. Wijnsma, Susan T. Veissi, Sem de Wijs, Thea van der Velden, Elena B. Volokhina, Frank A. D. T. G. Wagener, Nicole. C. A. J. van de Kar, L. P. van den Heuvel

**Affiliations:** ^1^ Department of Pediatric Nephrology, Radboud Institute for Molecular Life Sciences, Amalia Children's Hospital, Radboud University Medical Center, Nijmegen, Netherlands; ^2^ Department of Laboratory Medicine, Radboud University Medical Center, Nijmegen, Netherlands; ^3^ Department of Dentistry-Orthodontics and Craniofacial Biology, Radboud Institute for Molecular Life Sciences, Radboud University Medical Center, Nijmegen, Netherlands; ^4^ Department of Development and Regeneration, University Hospital Leuven, Leuven, Belgium

**Keywords:** heme, hemopexin, STEC-HUS, TMA, HO-1

## Abstract

Shiga-toxin (Stx)-producing *Escherichia coli* hemolytic-uremic syndrome (STEC-HUS) is one of the most common causes of acute kidney injury in children. Stx-mediated endothelial injury initiates the cascade leading to thrombotic microangiopathy (TMA), still the exact pathogenesis remains elusive. Interestingly, there is wide variability in clinical presentation and outcome. One explanation for this could be the enhancement of TMA through other factors. We hypothesize that heme, as released during extensive hemolysis, contributes to the etiology of TMA. Plasma levels of heme and its scavenger hemopexin and degrading enzyme heme-oxygenase-1 (HO-1) were measured in 48 STEC-HUS patients. Subsequently, the effect of these disease-specific heme concentrations, in combination with Stx, was assessed on primary human glomerular microvascular endothelial cells (HGMVECs). Significantly elevated plasma heme levels up to 21.2 µM were found in STEC-HUS patients compared to controls and were inversely correlated with low or depleted plasma hemopexin levels (R^2^ −0.74). Plasma levels of HO-1 are significantly elevated compared to controls. Interestingly, especially patients with high heme levels (n = 12, heme levels above 75 quartile range) had high plasma HO-1 levels with median of 332.5 (86–720) ng/ml (p = 0.008). Furthermore, heme is internalized leading to a significant increase in reactive oxygen species production and stimulated both nuclear translocation of NF-κB and increased levels of its target gene (tissue factor). In conclusion, we are the first to show elevated heme levels in patients with STEC-HUS. These increased heme levels mediate endothelial injury by promoting oxidative stress and a pro-inflammatory and pro-thrombotic state. Hence, heme may be a contributing and driving factor in the pathogenesis of STEC-HUS and could potentially amplify the cascade leading to TMA.

## Introduction

Renal thrombotic microangiopathy (TMA) is a heterogeneous group of disorders, characterized by vascular occlusion of capillaries due to thrombus formation, leading to thrombocytopenia, hemolytic anemia, and acute kidney injury (1, 2). In children, the most common cause of TMA is a gastro-intestinal infection with Shiga-toxin (Stx)-producing *Escherichia coli* (STEC) (2, 3). Up to now, it is believed that Stx, an AB toxin, as produced by STEC is the most important factor resulting in the development of hemolytic-uremic syndrome (HUS). However, only approximately 15% of all pediatric patients with STEC infection develop HUS, suggesting that also other factors are involved (4).

The exact pathogenesis of STEC-HUS is only partially understood (5). Stx can enter the bloodstream and bind to a receptor, the glycolipid globotriaosylceramide (Gb_3_) present on endothelial cell surfaces. After the uptake of the A subunit of Stx into the cell, cellular activation and damage is caused together with the promotion of a pro-inflammatory and pro-thrombotic state, leading to TMA (6). Yet, there is wide variability in clinical presentation and outcome in STEC-HUS ranging from no chronic sequelae to hypertension, proteinuria, end stage renal disease, or even death (2). One possible explanation for this broad range of clinical outcome could be the enhancement of TMA through additional factors causing endothelial damage.

One of these factors could be heme, as released during extensive hemolysis such as present in STEC-HUS patients (7). Free heme is known for its noxious effects and primarily causes oxidative and inflammatory stress, together with a pro-thrombotic effect (7–10). Furthermore, it has the unique ability to intercalate in the membrane of red blood cells, thereby enhancing hemolysis and heme accumulation.

Protection against free heme-mediated oxidative stress and inflammation includes scavengers, specific efflux transporters, and the heme-degrading enzymes heme-oxygenase (HO) (11). Following hemolysis, free vascular hemoglobin is captured by its scavenger haptoglobin. When haptoglobin is depleted hemoglobin gets converted to methemoglobin and releases its heme molecules (12). Free vascular heme is bound to the plasma proteins hemopexin or albumin, which transport it to the liver for degradation (13, 14). However, when large amounts of free heme and hemoglobin (locally) accumulate, like in a blood clot or after vascular deposition in the glomeruli, the heme/hemoglobin scavengers get overwhelmed or are unable to reach them (13, 15, 16).

In the absence of haptoglobin and hemopexin, already small amounts of free heme (or bioreactive heme) can be highly toxic and activate Toll-like receptor (TLR) 4-induced inflammatory signaling and cellular injury (17, 18). In addition, when heme proteins get trapped in a thrombus, as abundantly present during TMA, this can lead to accumulation of heme proteins resulting in heme levels as high as 350 µM (19). Moreover, circulating scavengers cannot reach the heme proteins when trapped in thrombi, leaving intracellular HO-1 as the sole protective mechanism (9, 20–22). In a HO-1 deficient patient, serum heme levels rose to 500 µM, which was accompanied with various oxidative and inflammatory complications (23). Unfortunately, this protective mechanism against heme could also be hampered in patients with STEC-HUS. Intriguingly, Bitzan et al. described the ability of Stx to inhibit HO-1 expression in human renal carcinoma-derived tubular epithelial cells (24). To our knowledge, no studies have been published regarding the effect of heme and Stx2 on HO-1 regulation in primary glomerular endothelial cells.

In a wide variety of diseases such as sickle cell disease, malaria, sepsis, and ischemia reperfusion injury heme has been described as culprit (17, 20, 25–28). Unfortunately, very little is known about the (patho)physiological levels of extracellular heme in case of the hemolysis seen in STEC-HUS patients. Knowledge regarding accurate and representative heme levels as present during hemolysis *in vivo* is necessary to study the possible noxious effect of heme levels *in vitro*. We hypothesize that extracellular accumulation of bioreactive heme can initiate and amplify the detrimental cascade leading to TMA. In this study we measured plasma levels of heme, hemopexin, and HO-1 in STEC-HUS patients, during the acute phase and assessed *in vitro* the effects of these disease specific heme concentrations, in combination with Stx2, on primary human glomerular microvascular endothelial cells.

## Methods

### Patient Cohort

All patients who presented in the Radboud University Medical Center (Radboudumc) Amalia Children’s hospital between 1990 and 2016 diagnosed with STEC-HUS were included. A clinical pattern of STEC-HUS was defined as; hemoglobin level below the lower limit of normal for the specific age and signs indicative of hemolysis, acute renal failure, thrombocytopenia <150 × 10^9^/L, and (bloody) diarrhea or family members with diarrhea. STEC-involvement was diagnosed by fecal diagnostics (culture, free fecal shiga-toxin assay, and/or PCR) and serology (for serotype O157). Both plasma and serum were collected on admission (acute phase) and stored at −80°C. Clinical data were collected retrospectively from medical records. In addition, 24 plasma samples of healthy adult controls and 26 samples of age matched controls were included after informed consent was obtained. Exclusion criteria for this control group were: fever, bacterial/viral infection in the past few weeks, chronic illness, inborn or acquired hematological / immune disorders, and the use of immunosuppressive drugs. Informed consent was given by the ethics committee (CMO Arnhem-Nijmegen) (2017–3490).

### Colorimetric Heme Quantification Assay

Plasma heme was measured using 1-Step™ Turbo 3,3',5,5'-Tetramethylbenzidine (TMB) enzyme-linked immunosorbent assay (ELISA) Substrate (Thermofisher scientific). This method is based on the oxidation of TMB by the pseudoperoxidase activity of heme. For this assay, hemin chloride porcine (designated as heme, Sigma-Aldrich) was diluted in 0.1 M NaOH (Merck) and the pH was adjusted to 7.8–8.2 (29). Subsequently, this solution was filtered through 0.2 µm pore size membrane (Whatman). Hereafter, serial dilutions (4.0–2.0–1.5–1–0.5–0.25–0 µM) of a 2 mM heme stock were made in 20 mM HEPES (Sigma-Aldrich) + 1% bovine serum albumin (BSA, Millipore), pH 7.4. Next, plasma samples (patients 10×, controls 2.5×) were diluted in 20 mM HEPES + 1% BSA, pH 7.4, where after 20 µl of every sample and 80 µl of the turbo TMB substrate was added to a microtiter plate (Greiner Bio-One™) and incubated for 10 min in the dark. The reaction was stopped with 100 µl 2 M sulfuric acid (Sigma-Aldrich) and absorbance was read at 450 nanometer (nm) with Victor 3 V multilabel plate reader (PerkinElmer).

### Hemopexin, Heme-Oxygenase 1, Hemolysis- and Icterus-Index Measurement

Hemopexin was measured in a sandwich ELISA. A microtiter plate (Greiner Bio-One™) was coated with 500 ng/ml anti-human hemopexin capture antibody (Bioporto) and incubated at 4°C overnight. Subsequently, standard dilutions of hemopexin from human plasma (0–50 ng/ml, Sigma-Aldrich) as well as diluted patient samples were added to the plate. Next 100 ng/ml of the detection antibody biotinylated anti-human hemopexin (Bioporto) was added to the plate followed by 1:1,000 dilution of streptavidin-HRP (RPN1231V GE healthcare). Subsequently, TMB Substrate (Sigma-Aldrich) was added and the absorbance was measured at wavelength of 450 nanometer (nm) with Victor 3 V multilabel plate reader (PerkinElmer).

Plasma HO-1 levels were determined by a sandwich ELISA using the human HO-1 matched antibody pair kit (Abcam) according to the manufacturer’s instructions.

Hemolysis and icterus index (respectively H-index and I-index) were determined using Serum Index Gen. 2 assay according manufacturer’s instructions (Cobas c system, Roche Diagnostics). H-index was used as marker for free hemoglobin. I-index was used as marker for bilirubin (both conjugated and unconjugated). H-index and I-index were determined based on absorbance measurements (570 and 480 nm primary wavelength and 700 and 505 nm secondary wavelength respectively) and expressed as µmol/L.

### Cell Culture 

Human glomerular microvascular endothelial cells (HGMVECs) were isolated and cultured as described previously by Van Setten et al. (30).

### Stimulation of HGMVECs 

Cells were cultured 24 h prior to the experiments in serum free conditions to avoid the effect of serum in experiments. For RNA and protein experiments, HGMVECs were seeded on gelatin coated six-well tissue culture plate (Corning® Costar®) at a density of 5 × 10^5^ cells and grown to confluent monolayers. HGMVECs require inflammatory mediator tumor necrosis factor-α (TNFα) to upregulate the Gb3 receptor on their cell surface (30, 31). Therefore, in the conditions where the effect of Stx2 in the presence or absence of heme was assessed, HGMVECs were preincubated with 10 ng/ml TNFα (Sigma-Aldrich) for 24 h at 37°C with 5% CO_2_. Subsequently, 100 pM Stx2 (Phoenix lab) was added to HGMVECs and incubated for 8 h at 37°C with 5% CO_2_. Next, 25 and 50 µM heme was added and incubated for additional 16 h at 37°C with 5% CO_2_.

### Quantitative Polymerase Chain Reaction (qPCR)

The total RNA was extracted using TRIzol reagent (Ambion life technologies) and RNA isolation kit according to the manufacturer’s instructions (NucleoSpin® RNA II from Macherey-Nagel). From the total RNA, 200 ng served as a template for cDNA synthesis in a reaction using a mix consisting of milliQ, reverse transcriptase buffer 5x (RT buffer 5x; Invitrogen), random primers (Promega), oligo dT (promega), dNTP’s, dithiothreitol (DTT; Invitrogen), recombinant RNasin® ribonuclease inhibitor (Invitrogen), Moloney Murine Leukemia Virus Reverse Transcriptase (M-MLV RT; Invitrogen). Primer efficiency of β-actin and GUSB were determined by making a serial dilution of cDNA (1–0.5, 0.25, 0.125, 0.0625, 0.031, 0.016 µg). Efficiency for the each primer was calculated by using the slope value. The efficiency for β-actin was 77.6% and for GUSB 112%. Finally, to confirm the specificity of each primer, the products were run in agarose gel. For qPCR analyses, a SYBR® Green master mix including primers (respectively forward and reverse primers for beta glucuronidase (GUSB) were 5’- AGA GTG GTG CTG AGG ATT GG-3’ and 5’-CCC TCA TGC TCT AGC GTG TC’-3; HMOX1 5’- AGA CAC CCT AAT GTG GCA GC-3’ and 5’- CTG AGC CAG GAA CAG AGT GG-3’; glutathione peroxidase 4 (GPX4) 5’- GCT GGA CGA GGG GAG GA-3’ and 5’- GAA GCC CCG GTA CTT GTC C-3’) was made. qPCR was performed using the Bio-Rad CFX96™ Real-Time PCR Detection System. Experiments were performed in duplicate and the values were normalized to GUSB using Δ-Δ Ct method (32).

### Western Blot

Protein was isolated with standard RIPA buffer [0.15 M NaCl (Sigma-Aldrich), 0.012 M Sodium Deoxycholate, 0.1% nonidet P-40 (NP40), 0.1% SDS (Sigma-Aldrich), 0.05 M Tris (Sigma-Aldrich) pH 7.5 and protease inhibitor cocktail (Sigma-Aldrich)]. The concentration of extracted protein was determined with BCA protein assay (Thermofisher Scientific) according to the manufacturer’s protocol. For HO-1 protein expression, 1 ug/ml protein was boiled in Laemmli buffer (Biorad). Sodium dodecyl sulfate polyacrylamide gel electrophoresis (SDS-PAGE) analysis was carried out using 10% resolving gels and 4% stacking gels. Subsequently, proteins were transferred to polyvinylidene difluoride (PVDF; Merck) membranes. Blots were blocked with PBS-5% skim milk (Merck) for an hour and incubated with primary antibodies, 2 µg/ml rabbit anti-heme-oxygenase 1 polyclonal antibody (Enzolifesciences) or 0.1 µg/ml anti- β-actin (Novus biological) diluted in PBS-5% BSA for 1 h at RT. Proteins were detected with 0.25 µg/ml HRP-conjugated goat-anti rabbit IgG antibody (Dako) diluted in PBS-5% BSA. Blots were developed using SuperSignal West Pico Chemiluminescent substrate according to the manufacturer’s instructions (Thermofisher scientific). The visualization of the bands was done with Biorad image lab software (Biorad). Semiquantitative analysis of the blots was performed with Image J software and the ratios of HO-1 and β-actin were plotted as fold changes.

### Immunofluorescence Imaging

5 × 10^4^ HGMVECs/well were seeded on 1% gelatin (Fluka) coated round glass coverslips Ø 1 cm (VWR) and grown to confluent monolayer. Subsequently, HGMVECs were stimulated with 25 or 50 µM heme and/or Zn(II) Mesoporphyrin IX (ZnMP; Frontier scientific) for 6 h. After stimulation, cells (n = 1 for ZnMP, and n = 3 for NF-κB) were fixed with 4% paraformaldehyde (PFA; Sigma-Aldrich) for 15 min at RT and subsequently stained with appropriate antibodies, 2 µg/ml of CD31 (mouse IgG1; Sanquin) and 4 µg/ml of monoclonal mouse α-human NF-κB p65 (F-6; Santa Cruz)) diluted in PBS-10% goat serum for 1 h at RT. Subsequently, 4 µg/ml of secondary antibody Alexa fluor goat α-mouse IgG (Thermofisher scientific) and the nuclear stain DAPI 1:1,000 diluted in PBS was added and incubated for 1 h at RT. Hereafter, the cells were washed three times with PBS. Finally, stained cells on the coverslips were mounted on microscopic slides (Thermofisher scientific). Slides were viewed with a Zeiss fluorescence microscope or by Olympus FV1000 confocal microcopy. To present the data of the multiple experiments (n = 3) performed for NF-κB translocation, a representative slide was chosen to incorporate in the figure.

### Reactive Oxygen Species (ROS) Measurement

The production of intracellular ROS was measured using the fluorescence probe reagent CM-H_2_DCFDA (Thermo Scientific) as described previously by Wilmer et al. (33). Briefly, confluent monolayers of HGMVECs (passage 8, 9, 13) on gelatin coated 96-well tissue culture plate were stimulated with 25 and 50 µM heme (prepared from 10 mM stock solution) or 50 µM hydrogen peroxide (H_2_O_2_; Merck). ROS formation was measured by fluorometry (excitation/emission 464/530 nm) after 1 h at 37°C with the Victor 3 V Multilabel Plate reader.

### Tissue Factor (TF) Measurement

With a cell-based ELISA, TF expression on the surface of HGMVECs was assessed. Confluent monolayer of HGMVECs (10.000 cells/well) on gelatin coated 96-well tissue culture plate (Corning® Costar®) were stimulated with 25 and 50 µM heme for 6 h. Subsequently, the cells were fixed with 0.025% glutaraldehyde (Merck), where after cells were incubated with biotinylated goat anti-human coagulation factor III detection antibody (DuoSet ELISA, R&D systems) diluted in M199-10% FCS according to the manufacturer’s instructions. Next, diluted streptavidin-HRP (DuoSet ELISA, R&D systems) was added. Signal was visualized with TMB (Sigma-Aldrich) substrate at 450 nm using Viktor 3 V multilabel plate reader.

### Flow Cytometric Apoptosis Assay

For assessment of apoptosis, approximately 1 × 10^5^ HGMVECs/well were seeded on gelatin (fluka) coated 24-well tissue culture plate (Corning® Costar®). The cells were stimulated with 25 and 50 µM heme prepared from 10 mM stock solution for 24 h. Subsequently, cells were detached with 0.25% Trypsin-EDTA (Gibco™) and collected into 1.5 ml Eppendorf tubes (Eppendorf). The cells were taken up in Annexin V-buffer and stained with FITC-labeled Annexin V and propidium iodide (Sigma-Aldrich) according to the manufacturer’s protocol (BioVision). All samples were analyzed on a FC500 flow cytometer with CXP software (Beckman Coulter). The flow cytometry data were analyzed with Kaluza Flow Analysis Software 1.3 (Beckman Coulter).

### Statistical Analysis

For *in vitro* experiments (i.e. ROS production, apoptosis and necrosis assay, ferroptosis, TF measurement) mean with standard deviation (SD) was calculated from repetitive experiments. T-test was used to compare data.

Patient characteristics and *in vivo* measurements (i.e. heme, hemopexin, HO-1 in plasma) are expressed as valid percentages for categorical variables and as median and 25–75 interquartile range (IQR) for continuous variables. Correlation coefficient was calculated using spearman correlation (R_s_), since data was not normally distributed. Regression analyses, with coefficient of determination R^2^, was performed to determine degree of correlation between two variables. Chi-square test was performed to compare categorical data and Mann-Whitney-U-test was used to compare continuous data. Heme levels were categorized with quartile ranges. In case a value was above the 75 IQR, the level was classified as high heme level.

P values less than 0.05 were considered statistically significant and 95% confidence intervals were reported when applicable. Statistical significance was reported in figures with use of these signs and correspond to p-value; * p < 0.05, ** p < 0.01, *** p < 0.001. All graphs were performed using GraphPad Prism software version 5. For statistical analyses, SPSS software (version 25.0) was used.

## Results

### Patient Characteristics

In total, 48 patients were diagnosed with STEC-HUS from 1990 to 2016 in the department of Pediatric Nephrology of Amalia Children’s Hospital Radboudumc of which medical information was present. Patients characteristics on admission are shown in **Table 1**. Patients showed signs of (mechanical) hemolytic anemia, based on low hemoglobin and increased lactate dehydrogenase (LDH) levels with decreased and often depleted haptoglobin levels. In total, 36 (75%) patients required dialyses.

**Table 1:** Patient characteristics.

Parameters	STEC-HUS (n = 48)
Age at disease onset, in years	3 (1–4)
Female	50%
Time between first day of illness and day of admission at academic hospital, in days	7 (5–8)
**Clinical presentation**	
Diarrhea	98%
*Of which bloody diarrhea*	75%
Fever defined as >38.2 Celsius	29%
Blood pressure ≥95 percentile for age and height	55%
Multi-organ involvement	31%
Duration hospital admission, in days	16 (11–21)
**Biochemical evaluation at presentation** * (reference range)*	
Hemoglobin (mmol/L) *(6.0–9.0)*	5.5 (4.6–6.0)
Platelets (× 10^9^/L) *(210–430)*	47 (32–69)
Haptoglobin (g/L) *(0.3–1.6)*	0.1 (0.1–0.14)
Leukocytes (× 10^9^/L)* (5.0–13.0)*	15 (11–22)
eGFR (ml/min/1.73m^2^) *(>90*)	11 (8–19)
LDH (U/L) *(<250)*	4,255 (2,609–6,290)
**Treatment**	
Dialysis	75%
Duration dialysis, in days	10 (7–15)
Need for erythrocyte transfusion	86%
Proven STEC infection	85%

Results show percentages or median with interquartile range (IQR). eGFR, estimated glomerular filtration rate; HUS, hemolytic-uremic syndrome; LDH, lactate dehydrogenase; n, number of patients of which data was available; STEC, Shiga-toxin-producing Escherichia coli.

### Heme and Its Scavengers in STEC-HUS Patients

**Figure 1:**
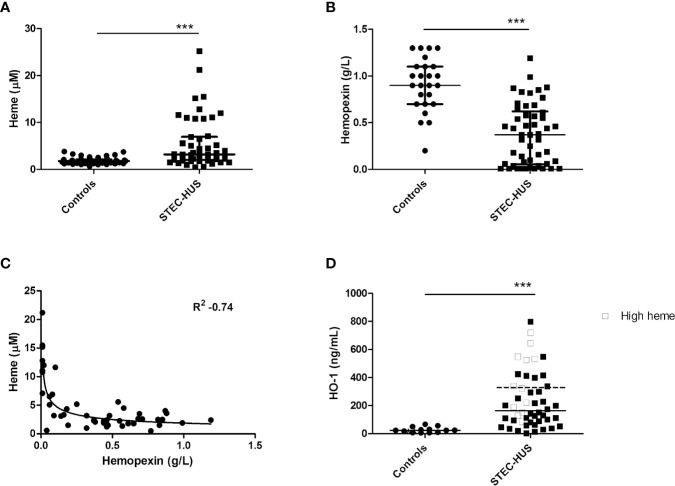
Plasma levels of heme, hemopexin, and HO-1 in STEC-HUS patients. **(A)** Significant higher heme levels (median with IQR) were observed in STEC-HUS patients (n = 48) compared to healthy controls (n = 24 adults and n = 26 age matched controls) (p < 0.001). **(B)** Significant lower hemopexin levels (median with IQR) were observed in STEC-HUS patients compared to healthy controls (n = 25) (p < 0.001). **(C)** Heme levels in STEC-HUS patients inversely correlated with plasma hemopexin levels (R_s_ 0.53, R^2^ −0.74, p < 0.001). **(D)** HO-1 levels (median) in plasma were significantly elevated in STEC-HUS patients (n = 48) compared to healthy controls (n = 13) (p < 0.001). Patients with high heme levels [n = 12, heme levels above 75 quartile range (open squares), median HO-1 level of these patients is represented by the dotted bar] had significantly higher plasma HO-1 levels (p = 0.008). ***p < 0.001; HO-1, heme-oxygenase-1; STEC-HUS, Shiga-toxin-producing *Escherichia coli* hemolytic-uremic syndrome.

Plasma levels of heme in STEC-HUS patients in acute phase were significantly higher (p < 0.0001) when compared to healthy controls. A median (range) of 3.2 (0.5–21.2) µM heme was found in STEC-HUS patients compared to 1.8 (0.6–3.8) µM in healthy controls (**Figure 1A**). Of note, no difference between pediatric and adult controls was observed. In total, 12 (25%) patients had heme levels above the 75-quartile range (6.3 µM) up till 21.2 µM. Median (IQR) time between hospital admission and sample collection was 3 (1–11.3) days. Furthermore, when samples were collected later on in the disease course, the risk of measuring lower heme levels was OR of 0.79 (95 % CI 0.64–0.99, p = 0.49).

STEC-HUS patients in the acute phase had significantly lower plasma hemopexin levels compared to healthy controls with median (range) of 0.4 (0.01–1.2) g/L and 0.9 (0.2–1.3) g/L respectively (p < 0.0001, **Figure 1B**). Hemopexin was depleted (<0.1 g/L) in 15 patients with a heme median (IQR) of 11 (6.5–12.8) µM. Moreover, a strong inverse correlation between measured heme levels and hemopexin levels was observed with R^2^ of −0.74 (**Figure 1C**). Of the 12 patients with high heme levels, nine had depleted hemopexin (<0.01 g/L), and the remaining three patients had hemopexin levels below 0.1 g/L.

### Influence of Stx2 on HO-1 Levels

**Figure 2:**
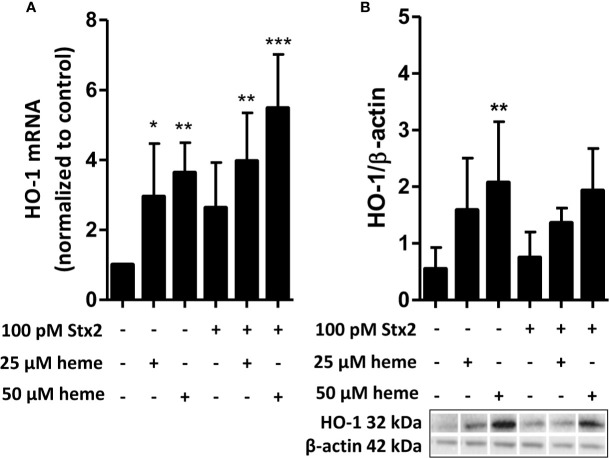
Stx2 does not decrease expression of the heme-degrading enzyme HO-1. **(A)** HGMVEC (passage 6, 6, 13) showed significantly upregulated HO-1 mRNA levels in response to 25 or 50 µM heme, which was, surprisingly, amplified by the co-presence of 100 pM Stx2. The mRNA expression of HO-1 was plotted as relative value to the expression of housekeeping gene GUSB. **(B)** Western blot analysis of HO-1 showed that the co-presence of 100 pM Stx2 did not reduce HO-1 protein levels. All experiments were performed in triplicate (n = 3). *p < 0.05, **p < 0.01, ***p < 0.001 GUSB, β-glucuronidase; HGMVEC, human glomerular microvascular endothelial cells; HO-1, heme-oxygenase-1; mRNA, messenger ribonucleic acid; Stx2, Shiga-toxin 2.

HO-1 is the most important intracellular heme-degrading enzyme and provides resistance to heme-induced oxidative and inflammatory stress and cell injury. Stx2 has previously been shown to enter endothelial cells by its receptor and interfere with protein translation. We therefore assessed HO-1 regulation in endothelial cells in response to heme in the presence or absence of Stx2 both on mRNA and protein level. HO-1 mRNA expression was found upregulated in HGMVECs in response to 25 or 50 µM heme after 6 h (**Figure 2A**). Interestingly, in the co-presence of Stx2, HO-1 mRNA upregulation was even more pronounced. Protein expression analysis shows that HO-1 is expressed regardless of the co-presence of Stx2 (**Figure 2B**). Altogether, these data indicate that both HO-1 mRNA and protein expression is upregulated in response to heme, regardless of the presence of Stx2.

This was in line with HO-1 levels in plasma, which were significantly elevated in STEC-HUS patients with median (range) of 163 (2.57–789) ng/ml compared to 23.7 (6–70) ng/ml in healthy controls (p < 0.001). Interestingly, especially patients with high heme levels (n = 12, heme levels above 75 quartile range) had high plasma HO-1 levels with median of 332.5 (86–720) ng/ml (p = 0.008, **Figure 1D**).

### Endothelial Cells Internalize Heme

**Figure 3:**
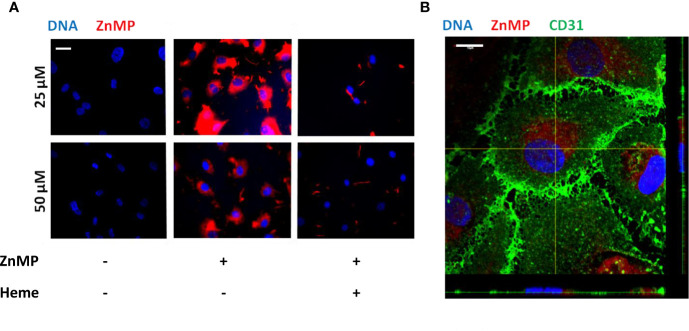
Endothelial cells internalize heme. **(A)** Exposure of HGMVEC (n = 1, passage 5) to 25 (top middle panel) or 50 (bottom middle panel) μM red-fluorescent ZnMP for 4 h yields increased signal intensity (200× magnification), which is decreased when same concentrations of heme is simultaneously added to cell culture (right top and bottom panels). **(B)** Confocal microscopy (HGMVEC passage 5, 200× magnification) confirms endothelial uptake of red-fluorescent ZnMP. Scale bars: 10 μm. HGMVEC, human glomerular microvascular endothelial cell; ZnMP, zinc mesoporphyrin; CD31, endothelial cell marker (green).

As heme levels can be elevated in STEC-HUS patients, we next aimed to characterize the functional consequences of disease-relevant heme levels on primary HGMVECs. Exposure of HGMVECs to 25 and 50 μM auto-fluorescent heme analog, ZnMP caused increased signal in the cells, with no reciprocal difference between both concentrations (**Figure 3A**, middle panels). To confirm whether heme and not only its analogue ZnMP is internalized by endothelial cells, endothelial cells were next exposed to 25 and 50 μM ZnMP and heme simultaneously. A decreased ZnMP signal in endothelial cells was observed due to competitive uptake of ZnMP and heme (**Figure 3A**, right panels). Finally, to confirm that ZnMP was truly internalized by the endothelial cells rather than sticking to its outer membrane, the ZnMP signal in endothelial cells by confocal microscopy was established. By co-staining the outer membrane with CD31, we confirmed that the ZnMP signal was indeed within the HGMVEC (**Figure 3B**). In sum, these data indicate that human glomerular endothelial cells have the capacity to internalize heme.

### Heme Causes Oxidative and Inflammatory Stress and Promotes a Pro-Thrombotic State 

**Figure 4:**
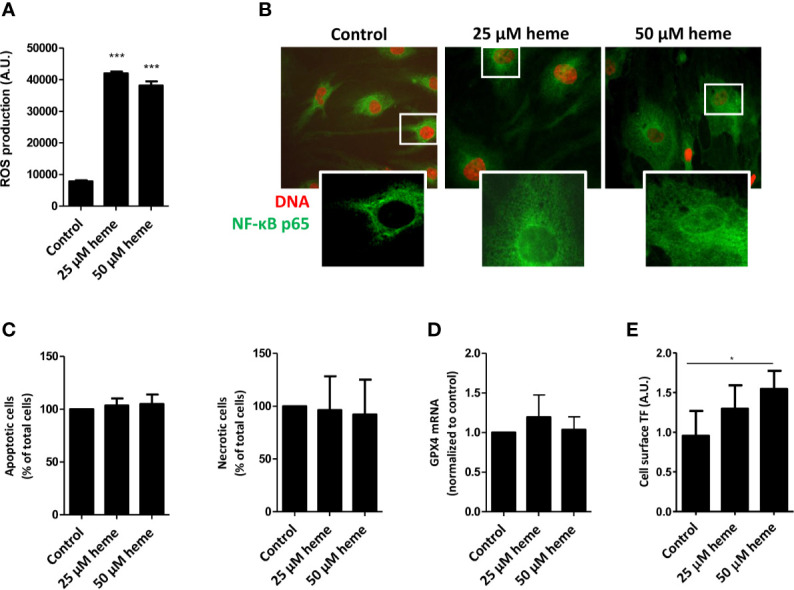
Heme causes endothelial cell damage and promotes pro-thrombotic state. **(A)** Exposure HGMVEC (n = 3, passage 8, 9, 13) to 25 or 50 μM heme results in significantly increased reactive oxygen species (ROS) generation. **(B)** HGMVEC (passage 5) exposed to 25 or 50 µM heme show nuclear translocation of NF-κB p65 (200× magnification). **(C)** HGMVEC (n = 3, passage 6, 6, 7) exposed to 25 or 50 μM heme for 24 h does not result in an increase in apoptosis or necrosis compared to the control condition. **(D)** HGMVEC (n = 3, passage 6, 6, 13) stimulated with 25 or 50 µM heme does not cause ferroptosis. **(E)** HGMVEC (n = 3, passage 5, 6, 8) exposure to 25 and 50 µM heme induces protein expression of the NF-κB responsive target gene membrane-bound tissue factor. *p < 0.05, ***p < 0.001, compared to control unless specified otherwise. Scale bars: 10 μm. HGMVEC, human glomerular microvascular endothelial cell; ROS, reactive oxygen species; AU, arbitrary units.

To assess whether the exposure of endothelial cells to disease-relevant heme levels causes endothelial cell activation, cell stress, and cell death, we first analyzed reactive oxygen species (ROS) production by HGMVECs either in the absence or presence of heme. Treatment of HGMVECs with heme (25 or 50 µM) resulted in significantly increased ROS generation after 1 h (**Figure 4A**). Furthermore, heme-induced ROS generation led to a profound nuclear translocation of the pro-inflammatory transcription factor NF-κB p65 subunit (**Figure 4B**). HGMVECs exposed to heme (25 or 50 µM) for 24 h did not show increased apoptosis or necrosis (**Figure 4C**). Ferroptosis-inducing compounds converge often on GPX4 inhibition. Although heme-mediated cell death is often associated with ferroptosis, we did not observe ferroptosis in response to 25 or 50 µM heme after 16 h (**Figure 4D**). As the pro-thrombotic state, present during TMA caused by STEC infection, is an important feature of STEC-HUS, we assessed the effect of heme on NF-κB responsive target gene tissue factor (TF), the key initiator of the coagulation cascade using ELISA. A significantly increased cell surface TF protein expression was observed on HGMVECs following incubation with 25 or 50 µM heme, further supporting that heme not only increases oxidative and inflammatory stress, but may also enhance the pro-thrombotic state in STEC-HUS patients (**Figure 4E**).

### Discussion 

Although STEC-HUS is one of the most common causes of acute kidney injury in children, the exact pathogenesis and the high diversity in clinical severity is only partially understood. In general, extensive hemolysis leads to release of the noxious molecule heme. To our knowledge, we are the first to show elevated heme levels in patients with STEC-HUS. These increased levels of extracellular heme are associated with depleted hemopexin and increased expression of HO-1 in plasma, suggesting cellular damage, oxidative and inflammatory stress. *In vitro*, primary HGMVECs exposed to heme resulted in ROS production and stimulation of both a pro-inflammatory (e.g. nuclear translocation of NF-κB p65 subunit) and a pro-thrombotic state (e.g. increased TF expression). Yet, it seems that the cytoprotective effects of HO-1 against the noxious effects of heme are not affected by the tested levels of Stx2. Heme released during mechanical hemolysis and cellular injury could contribute, amplify, and even drive the detrimental cascade leading to TMA.

Previous literature regarding pathogenic heme levels refer mostly to one article of Muller-Eberhard et al. published in 1968 (13). As one of the first, they described free heme levels, determined with the use of the pyridine hemochromagen assay, in patients with various hemolytic diseases in correlation with hemopexin levels (13). From 6 µg/ml (˜10 µM) of heme onwards hemopexin levels were depleted, and heme levels up to 30 µg/ml (˜50 µM) were measured (13). Since then, accumulating studies demonstrate that heme can contribute to the etiology of a wide variety of diseases as exemplified below (20, 34). Some studies report heme levels, mainly measured in patients with sickle cell disease, where heme levels around 4 (up to 20) µM were measured (35). Heme was found to strongly contribute to the pathogenesis of sickle cell disease, whereas hemopexin and HO-1 protect against heme-induced detrimental effects (18, 36, 37). Reported heme levels in patients with severe malaria are similar with median of 10 (IQR 4–22) µM and strongly contribute to its pathogenesis (26, 38). Similarly, heme has been shown to augment the severity of sepsis (27). These previously reported heme values correspond well with the values we found in STEC-HUS patients. Most likely the systemic heme levels as measured are an underestimation of heme levels within the glomeruli, where, in the presence of thrombi, heme proteins accumulate, obstructing haptoglobin and hemopexin access.

Heme easily reacts with various proteins, with the highest affinity for hemopexin, but is also known to bind to other proteins like albumin and lipoproteins (21, 22). However, only hemopexin and HO-1 are capable of fully neutralizing the noxious effect of heme, so in case heme is bound to for example albumin it remains partly bioreactive (39). To measure these bioreactive levels of heme, we used an assay which takes advantage of the Fenton reaction of heme. When bioreactive heme comes in contact with the TMB substrate, this is oxidized resulting in a color which can be spectrophotometrically quantified. This assay has been used previously to measure heme levels in different patient cohorts and has been proven simple and sensitive when compared to assays using for instance toxic substances such as pyridine (13, 29, 35, 38). The measured plasma heme levels in our study reflect most likely the circulating levels of bioreactive heme. The observed heme levels are supported by the inverse correlation between low hemopexin levels and measured heme levels in the plasma. Moreover, in 15 patients hemopexin measured in plasma in the acute phase was undetectable with a strong correlation with high heme levels. This is further supported by haptoglobin levels, used as marker of hemolysis, which was undetectable in almost all patients. Hereby making hemopexin a reliable marker for high heme levels. Subsequently, significantly elevated HO-1 levels were observed in STEC-HUS patients compared to the healthy adult controls. HO-1 is known to be upregulated in response to various triggers like ischemia, inflammation, and heme (39). However, in comparison to the well described extracellular scavengers like hemopexin, albumin, and lipoproteins (all measured in g/L), concentrations of extracellular HO-1 are considerably lower (ng/ml). Most likely, HO-1 mainly exhibits its protective function intracellularly, however, we cannot exclude that it acts as a heme scavenger in serum. The source of increased plasma HO-1 remains unclear, yet it is likely that HO-1 derives from injured and stressed cells releasing their content.

As previously proposed by Bitzan et al. Stx2 (100 pM) could inhibit HO-1 levels, when tested on human renal carcinoma-derived tubular epithelial cells (24). Since STEC-HUS is mainly a glomerular disease we assessed HO-1 expression in HGMVECs with co-stimulation of Stx2 and heme. The presence of Stx2 caused an upregulation of HO-1 on mRNA level. In contrast to Bitzan et al., HO-1 protein expression was not altered by Stx2. Most likely this could be explained by the low concentrations of Stx2 in our experiments as it was previously shown that 100 pM of Stx2 merely reduces protein synthesis in HGMVECs (30). However, higher concentrations of Stx2 have shown to inhibit protein synthesis of HO-1 and therefore could further enhance heme-induced cytotoxicity in STEC-HUS patients. Moreover, May et al. showed impaired HO-1 upregulation in the presence of heme in glomerular endothelial cells, compared to other (macrovascular) endothelial cells. This suggests that especially glomerular endothelial cells are highly susceptible for cytotoxic effects of heme (40).

Interestingly, individual differences exist in protection against heme. For example, due to a polymorphism at the promoter region of HO-1, there is a wide variety between individuals in their ability to upregulate HO-1 expression following a stimulus. Individuals with longer (GT) n repeats in their promoter region have reduced HO-1 activity and, as a consequence, are more susceptible to acute kidney injury. Haptoglobin polymorphisms also offer differential protection against hemolysis-derived hemoglobin because of different efficacy in binding and subsequently protection against inflammatory injury (41). Also polymorphisms in hemopexin have been found that could possibly have differential binding activity and therefore providing differential protection from heme (42). These differences in defense against heme could partly explain the individual variability in susceptibility to develop TMA (9, 11). For this purpose, it may be beneficial to screen individuals for HO-1, and hemopexin polymorphisms.

Various studies have assessed the effect of heme *in vitro*, however using a broad range of heme levels, up till 100 µM (24, 43). In our study, we used primary HGMVECs as these are the main target cells in the pathogenesis of HUS. By using a fluorescent heme analogue, ZnMP, we showed that heme is internalized by these endothelial cells. The mechanisms by which heme is internalized remains largely unknown. Various hypothetical theories of heme internalization have been proposed. First of all, heme has a highly lipophilic nature and may therefore intercalate into the hydrophobic phospholipid bilayer of cell membranes. Although we did not observe cellular injury upon exposure to relatively low amounts of heme, we previously demonstrated that heme can cause cellular injury and death (11, 21). A role for heme transporters is perhaps a more plausible explanation for the observed internalization and transport of heme (11, 44).

To assess the effect of disease specific heme levels we choose a concentration of 25 and 50 µM, taking into consideration our measurements of heme in our STEC-HUS cohort (levels up till 25 µM ) and values among others measured by Muller-Eberhard et al. (levels up till 50 µM) (13). When exposing HGMVECs to these heme levels, we observed increased ROS production together with induction of a pro-thrombotic and pro-inflammatory state. These results are in line with previous published studies (9). The generation of free radicals by the Fenton reaction is to date still considered the major form of ROS generation by heme and an important mechanism of heme-induced cytotoxicity. Moreover, we demonstrated that heme induced nuclear translocation of NF-κB, which is associated with upregulation of its target gene TF, which gets expressed on the surface of the endothelial cells, facilitating the formation of thrombi on the damaged endothelium. Hence, these observations could also be explained by interactions between heme and TLR-4 (15, 45). As TLR-4 is highly expressed on endothelial cells, and as ligation to TLR-4 does generally not result in receptor-mediated endocytosis but rather in activation of pro-inflammatory signaling pathways (e.g. the NF-ĸB nuclear translocation), it is plausible that the internalization of heme is not required for the observed (pro-inflammatory) events in endothelial cells. As shown by Belcher et al. heme activates TLR-4 signaling leading to vaso-occlusion due to degranulation of Weibel-Palade bodies and expression of vascular adhesion molecules in models of sickle cell disease (45, 46).

Measurement of heme levels is considered quite difficult. Using the TMB assay, our measured heme levels could be influenced by additional factors like free hemoglobin or bilirubin, also present during extensive hemolysis. However, free hemoglobin levels (measured using the hemolysis index; H-index) or icterus index (I-index; marker for high bilirubin levels) did not correlate with TMB results (see **Supplementary Figures 1A, C**). Haptoglobin is the scavenger of free hemoglobin, however in contrast to the strong correlation between hemopexin and heme, no correlation was found between haptoglobin and the presumed heme levels, indicating that the heme levels obtained with TMB seem specific for bioreactive heme (see **Supplementary Figure 1B**). Furthermore, of the 48 patients with TMA who were clinically suspected of STEC-HUS, in 85% evidence STEC infection was provided. It is known that STEC diagnostics can be difficult due to several factors, and it is important to combine fecal diagnostics with serology (47). In these patients only serotype O157 was detected with serology. The remaining 15% of the patients experienced no recurrence to our knowledge (indicative of atypical HUS) and other causes of TMA were ruled out, leaving STEC-HUS as most likely diagnosis.

As the production of ROS is implicated in pro-thrombotic (TF expression) and (pro-inflammatory) NF-κB-dependent signaling, it would be important to investigate whether ROS scavengers are able to prevent the TF expression or/and the nuclear translocation of NF-κB in heme-treated endothelial cells. Furthermore, in a murine model of STEC-HUS, the administration of anti-oxidants was shown to counteract important pathogenic events (e.g. platelet activation, renal damage) and ameliorate disease pathology (48). Thus, ROS scavengers may protect against the detrimental effects of heme in HUS.

Although STEC-HUS is merely treated symptomatically up till now, our findings suggest several potential future therapeutic candidates. One strategy could be to supplement the depleted scavengers like haptoglobin, to capture hemoglobin and prevents its decay into heme, or more naturally hemopexin to neutralize heme (49, 50). Various animal studies have been performed to look at the effect of therapeutic hemopexin administration with promising results (22, 51). In contrast to hemopexin, haptoglobin is already present as orphan drug. Hypothetically, by administrating haptoglobin, free hemoglobin would be bound and therefore could not be oxidized and release heme, hence no heme would be present. Although up till some extent damage is already caused at time of admission, most patients still have active disease with ongoing TMA at presentation.

In this study we focused on patients with STEC-HUS. Yet, we hypothesize that similar high heme levels could be observed in other patient cohorts like atypical HUS (aHUS). Atypical HUS is caused by overactivation and dysregulation of the complement system, resulting in TMA. It would be highly interesting to measure heme, hemopexin, and HO-1 plasma levels in patients with aHUS since various studies showed a strong effect of heme on complement deposition on endothelial cells. Hereby, indicating a key role of heme in amplifying complement overactivation on the endothelial level. Although the release of Stx remains the culprit in STEC-HUS, various studies reported secondary complement activation following STEC infection (52, 53). Therefore, it would be highly interesting to study the influence of heme on complement activation in patients with STEC-infection potentially further enhancing the susceptibility to develop TMA.

In conclusion, heme may be a contributing and driving factor in the pathogenesis of STEC-HUS and could potentially amplify the cascade leading to TMA. In this study we have provided evidence that elevated heme levels are present in STEC-HUS patients. Moreover, the observed heme levels have a strong inverse correlation with hemopexin levels, making hemopexin a reliable biomarker for toxic levels of heme present *in vivo*. Moreover, since these elevated heme levels promoted oxidative, pro-inflammatory and thrombotic stress *in vitro* we may develop novel strategies to target heme and attenuate disease.

## Data Availability Statement

The datasets on in vitro work generated for this study are available on request to the corresponding author. The datasets regarding patient data are not publicly available since at the time of requiring informed consent, patients did not give permission to publish specific data sets. Additional information is available from the corresponding author on reasonable request.

## Ethics Statement

Informed consent was provided by the participant and/or legal guardian/next of kin. Informed consent was provided by ethics committee.

## Author Contributions

KW collected and analyzed medical data, designed experiments, and co-wrote the manuscript together with SV. SV designed and performed the experiments, analyzed the data, and co-wrote the manuscript together with KW. SW collected medical data of included patients and critically reviewed and approved final report. TV performed the experiments and was involved in data interpretation. EV was involved in data interpretation and critically reviewed and approved final report. FW provided reagents related to the heme-HO system, was involved in data interpretation, critically reviewed and approved final report. NK was responsible for included patients, critically reviewed and approved final report. LH designed experimental setup, was responsible for the performed experiments, and critically reviewed and approved final report. All authors contributed to the article and approved the submitted version.

## Funding

This work was supported by the Sengers Stipendium, granted from the Foundation for Pediatric Research, of Radboudumc Amalia Childrens Hospital, to LH, NK, KW, and SV. ZonMw, “Goed Gebruik Geneesmiddelen” (project number: 836031008) to NK and Dutch Kidney Foundation (13OI116) to EV.

## Conflict of Interest

The authors declare that the research was conducted in the absence of any commercial or financial relationships that could be construed as a potential conflict of interest.

## Supplementary Material

Supplementary Figure 1 No correlation between free heme and free hemoglobin, haptoglobin or bilirubin levels. **(A)** Hemoglobin levels (µmol/L), measured with spectrophotometric assay measuring hemolysis index, reported as H-index, were determined in 48 patients with STEC-HUS and did not correlate with free heme (µM) measured with TMB (R_s_ 0.26, R^2^ 0.31). **(B)** No strong correlation was observed for haptoglobin levels compared to heme levels measured in 48 STEC-HUS patients (R_s_ 0.47, R^2^ 0.15). **(C)** Icterus-index (I-index), as masker for elevated bilirubin levels, measured with spectrophotometric assay was determined. No correlation between I-index and heme levels could be observed (R_s_ 0.031, R^2^ 0.021).
