# Supplementary figures and images for: Heme as Possible Contributing Factor in the Evolvement of Shiga-Toxin Escherichia coli Induced Hemolytic-Uremic Syndrome
Source: Front Immunol. 2020 Dec 22;11:547406. doi: 10.3389/fimmu.2020.547406 (PMC7783363; doi:10.3389/fimmu.2020.547406)

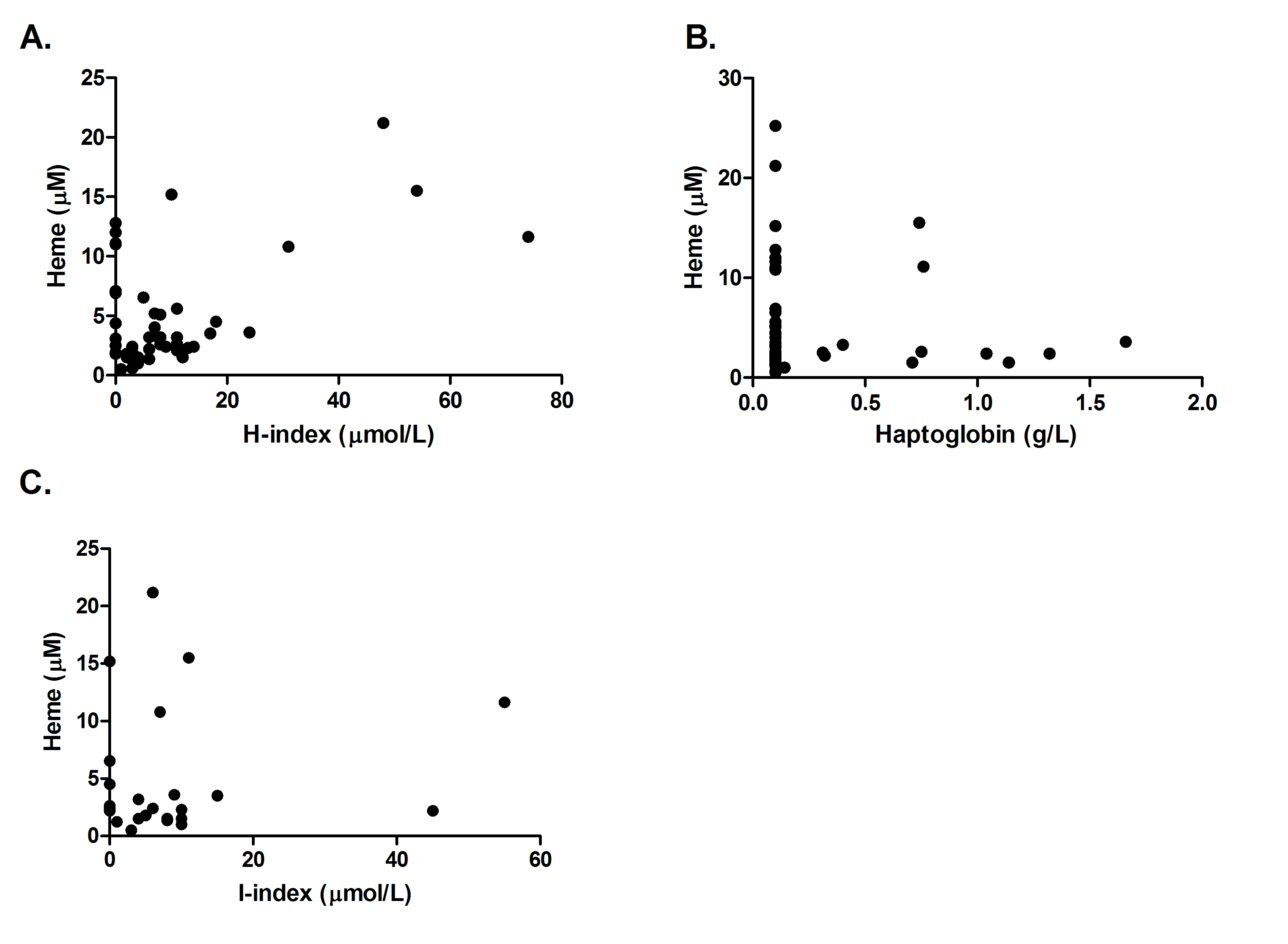

Supplement: Supplementary Figure 1 — No correlation between free heme and free hemoglobin, haptoglobin or bilirubin levels. (A) Hemoglobin levels (µmol/L), measured with spectrophotometric assay measuring hemolysis index, reported as H-index, were determined in 48 patients with STEC-HUS and did not correlate with free heme (µM) measured with TMB (Rs 0.26, R2 0.31). (B) No strong correlation was observed for haptoglobin levels compared to heme levels measured in 48 STEC-HUS patients (Rs 0.47, R2 0.15). (C) Icterus-index (I-index), as masker for elevated bilirubin levels, measured with spectrophotometric assay was determined. No correlation between I-index and heme levels could be observed (Rs 0.031, R2 0.021). [file Image_1.tif]
